# Activation of TRPC6 by AngⅡ Induces Podocyte Injury and Participates in Proteinuria of Nephrotic Syndrome

**DOI:** 10.3389/fphar.2022.915153

**Published:** 2022-08-03

**Authors:** Ye Feng, Manman Li, Yunlai Wang, Mo Yang, Gaoxiang Shi, Dengke Yin, Zihua Xuan, Fan Xu

**Affiliations:** ^1^ School of Pharmacy, Anhui University of Chinese Medicine, Hefei, China; ^2^ Anhui Province Key Laboratory of Chinese Medicinal Formula, Hefei, China

**Keywords:** nephrotic syndrome, podocytes, angiotensinⅡ, transient receptor potential cation channel 6, proteinuria

## Abstract

**Background:** Nephrotic syndrome (NS) is a common glomerular disease, and podocyte injury is the character of primary NS, usually caused by minimal change disease and membranous nephropathy. Podocytopathy is primarily associated with glomerular proteinuria. Losartan, an angiotensin receptor blocker (ARB), is commonly used in the treatment of NS, and the AngiotensinⅡ (AngⅡ)–transient receptor potential ion channel 6 (TRPC6) axis has been reported to act on podocytes to regulate proteinuria in NS. Therefore, the purpose of this study was to explore the relationship in between AngⅡ–TRPC6, podocyte injury, and proteinuria based on the adriamycin (ADR) NS rat model.

**Method:** All male rats were divided into three groups: control group, model group, and ARB group. The rats in the model group were induced by ADR, and the rats in the ARB group received losartan after induction of renal injury for 4 weeks. The changes in parameters related to renal dysfunction, and glomerular and podocyte structural damage, such as AngⅡ, AngⅡ type I receptor (AT1R), TRPC6, CaN, Caspase-3, Nephrin, and Podocin, were analyzed. Furthermore, the kidneys were isolated for study *via* transmission electron microscopy (TEM), immunohistochemistry, and western blot (WB) after the rats were sacrificed. *In vitro*, immortalized mouse MPC5 podocytes were used to investigate the regulatory effect of flufenamic acid (Flu) and SAR7334 (SAR) on the AngⅡ-TRPC6 signaling axis. Flow cytometry and WB were conducted to determine the relationship between podocyte injury and AngⅡ-TRPC6.

**Results:** *In vivo* results showed that NS rats developed massive albuminuria and abnormal renal function, accompanied by abnormally increased levels of AngⅡ, TRPC6, AT1R, and CaN and a decreased expression of actin molecules in podocytes, extensive fusion of foot processes (FP), loss of glomerular structural integrity, collapse of podocyte structure, and skeletal reorganization. *In vitro* experiments indicated that both AngⅡ and Flu (the specific agonist of TRPC6) stimulated the expressions of TRPC6, AT1R, and Caspase-3 in podocytes. The AngⅡ receptor–blocker losartan and TRPC6-specific inhibitor SAR blocked the overexpression of the aforementioned proteins. In addition, SAR also attenuated the degradation of podocyte structural proteins and inhibited the fluorescence intensity of intracellular calcium (Ca^2+^) and cell apoptosis.

**Conclusion:** The involvement of AngⅡ in the occurrence of NS proteinuria may be related to podocyte injury induced by activated TRPC6.

## Introduction

Nephrotic syndrome (NS) is a common clinical glomerular disease, and massive proteinuria is its main clinical indication (Singh et al., 2019). The main pathological manifestations of this disease are increased glomerular basement membrane permeability and decreased glomerular filtration rate (Miner, 2012). The glomerular filtration barrier (GFB) is mainly composed of three layers, namely, glomerular endothelial cells, basement membrane, and podocytes, respectively, from the inside to outside, and is an important molecular and charge barrier for maintaining normal filtration function of the kidneys. Podocytes are an important hub for maintaining the GFB (Yue et al., 2015; Forrester et al., 2018). It prevents macromolecular proteins carrying the same- charge properties from leaking out of the filtration barrier. This is the last barrier to proteinuria (Kawachi and Fukusumi, 2020). As the target cells of different degrees of renal injury, podocytes run through the whole process of occurrence and development of NS and so have become gradually recognized by people and are a research hotspot of proteinuria diseases.

AngiotensinⅡ (AngⅡ) is a strong vasoconstrictor active factor, which can induce a series of pathological reactions such as vasoconstriction, sodium and water retention, as well as pro-inflammation and pro-fibrosis on tissues (Maier et al., 2015; Fu et al., 2019a; Fu et al., 2019b; Jeong et al., 2019). When NS occurs, the body stimulates the renin–angiotensin–aldosterone system (RAS) and sympathetic nerve endings, resulting in proteinuria, thereby reducing the body’s blood volume and increasing the synthesis and secretion of AngⅡ (Yue et al., 2015). Clinical studies have shown that the level of AngⅡ in the renal tissue of patients with glomerulopathy is elevated and is involved in mediating inflammation and fibrosis in glomerulopathy (Beck et al., 2013). The protective effect of inhibiting the biological activity of AngⅡ has been confirmed, but the molecular mechanism in NS is still unclear (Koizumi et al., 2019).

Podocyte injury has been reported to be associated with intracellular calcium overload (Dryer et al., 2019). Calcium ions (Ca^2+^) act as a second messenger to regulate apoptosis, and NS has been found to cause calcium homeostasis imbalance (Beck et al., 2013). Transient receptor potential ion channel 6 (TRPC6), a nonselective downstream channel of Ca^2+^, has attracted more and more attention. TRPC6 is distributed in the glomerulus and is widely expressed in podocytes (Abrahamson, 1987). It is a structural protein involved in maintaining the structural stability of the podocyte skeleton and in regulating Ca^2+^ homeostasis (Cardoso et al., 2018; Shi et al., 2020). In addition, it has been confirmed by patch-clamp electrophysiology that the current of TRPC6 was significantly increased, leading to Ca^2+^ overload in podocytes, resulting in cell damage in proteinuria disease (Guo et al., 2014; Yu et al., 2018). Regardless of the results of clinical or experimental studies, TRPC6 is abnormally expressed in glomerular diseases (Yamazaki et al., 2016; Zhu et al., 2018; Dolinina et al., 2021). Therefore, TRPC6 may be a breakthrough point in elucidating the pathogenesis of NS. Most importantly, it was recently reported that AngⅡ-stimulated podocytes can significantly increase the expression of TRPC6 (Wang et al., 2021). Therefore, the AngⅡ-TRPC6 targeting pathway may be a new strategy for modern NS treatment. However, the interaction between AngⅡ and TRPC6 in the occurrence and development of NS podocyte injury remains unclear. Based on this, the present study intends to investigate whether AngⅡ can induce NS podocyte molecular barrier damage by mediating the abnormal activation of TRPC6 ion channels, further leading to proteinuria through animal and cell experiments, and explain the possible molecular mechanism.

## Materials and Methods

### Antibodies and Reagents

The primary antibodies against AT1R (R22571), Nephrin (503048) were purchased from ChengDu Zen BioScience Co., Ltd, China. The primary antibodies against cleaved Caspase-3 (ab32351), TRPC6 (ab233413), and Podocin (ab181143) were purchased from Abcam Company, Britain. The secondary antibodies horseradish peroxidase (HRP)–conjugated anti-mouse IgG (EF0002), β-actin (700068) were bought from Zhongshan Biotechnology Company, China. The BCA kit, Albumin kit (ALB, 1026A20), urea nitrogen (BUN, TC0567), creatinine (CR, TC0565), and total protein (TP, TC0543) were bought from Beijing Regen Biotechnology Company, China. Total cholesterol (TC, 201023) and triglyceride (TG, 201109) were got from Nanjing Jiancheng Biological Engineering Institute, China. Adriamycin (ADR, Doxorubicin Hydrochloride Injection, 130704) was bought from Zhejiang Hisheng Pharmaceutical Company, China. Losartan potassium tablets (T000046) were purchased from UK Merck Sharp & Dohme Ltd. The AngⅡ radioimmunoassay kit (JL11637) and CaN (JL12083) were bought from Shanghai Jianglai Biotechnology Co. Ltd., China. Fetal bovine serum (KC001) was purchased from Kel Inc., United States. The RPMI 1640 medium (CF0001) was got from Shandong Sparkjade Technology Instrument Company, China. SAR7334 (SAR, GC-33424) and Flufenamic acid (Flu, GC-13446) were got from MedChemExpress Company, America. CCK-8 (CT0001) was purchased from Shangdong Sparkjade Biotechnology Company, China. Beckman AU-5800 Automatic Biochemical Analyzer was purchased from Beckman Coulter, United States.

### Animal Handle

This study was conducted under the approval of animal ethics and supervised by Anhui University of Chinese Medicine Ethics Committee. All experiments were performed using male Sprague–Dawley rats weighing from 180 to 220 g, purchased from Jinan Pengyue Experimental Animal Breeding Co., Ltd., certificate number: SCXK (LU) 20190003. All rats were housed at 25 ± 1°C and 55 ± 5% humidity using a 12-h light/dark cycle and were given standard feed. After adaptive feeding for 1 week, the rats were randomly divided into three groups according to the random number table. The three groups were the control group, model group, and ARB group. The research group determined the injection method, dose, and frequency of the ADR nephrotic syndrome model through experimental screening in the early stage. The NS model was replicated by two injections (4 mg kg^−1^ in the first week, 2 mg kg^−1^ in the second week) of ADR into the rats’ tail veins; meanwhile, the control group was given the same amount of normal saline. Two weeks after ADR intervention, the rats were placed in metabolic cages and fasted to collect urine for 24 h, drinking water normally during the period. The 24-h urine protein content was measured by an automatic biochemical analyzer. If the 24-h urine protein content of the model group was significantly higher than that of the normal group, it was considered to have successfully replicated the rat model of NS. And then, the NS rats were randomly divided into the model group (given the same amount of normal saline) and the ARB group (losartan, 30 mg kg^−1^ d^−1^) (Fan et al., 2014; da Silva-Bertani et al., 2020). The rats in each group were given the corresponding drugs on the day of the successful modeling. The administration time was 4 weeks, during which the animals in each group were free to eat and drink.

### Podocytes Culture and Treatment

Immortalized mouse MPC5 podocytes were used in this experiment. The cell lines were purchased from Beijing Beina Chuanglian Biotechnology Institute, batch number: BNCC337685. Podocytes were cultured in an RPMI 1640 complete culture medium containing interferon γ, which consisted of 10% fetal bovine serum, 100 U/ml penicillin–streptomycin, and RPMI 1640 basic culture medium. The growth of the podocytes was promoted in an incubator at 33°C with 5% CO_2_ volume fraction. When the cell proliferation reached 80%, the cells were transferred to a complete culture medium RPMI 1640 without interferon γ and induced to mature in an incubator at 37°C. The cells were transferred to the sixth generation. When the area of the podocytes accounted for 40–50% of the culture flask, we used the optimized stimulation concentration of AngⅡ (10^−6^ mol L^−1^) to create the model. Twenty-four hours later, the cells were randomly divided into the AngⅡ, AngⅡ + SAR (10^−6^ mol L^−1^, 30 min), Flu (10^−4^ mol L^−1^, 2 h), and control groups without stimulation. The cells were cocultured with the drugs in each group, and the next detection was carried out.

### Biochemical of Blood and Urine

At the end of week 4 after the drug intervention, the body weight (BW) and urine protein of the rats were detected. Then, the rats were anesthetized and blood samples (5 ml) were drawn from the abdominal aorta. The biochemical parameters in the blood such as ALB, blood urea nitrogen (BUN), serum creatinine (SCR), TC, TG, and TP were tested. The serum and urine samples were measured using an automatic biochemical analyzer. The kidney hypertrophy index (KHI) was calculated according to the method described by Lane et al. (1992) that is KHI = kidney weight (KW)/BW (Pollak et al., 2014). Meanwhile, the urine albumin excretory rate (UAE) was expressed as the ratio of urinary albumin to urine creatinine.

### Light Microscopy Examination

For assessment by light microscopy, the tissue samples from the renal cortex were fixed with 4% paraformaldehyde solution, embedded in paraffin, cut into 3-μm-thick sections and stained with hematoxylin–eosin (HE). The slices were dehydrated and sealed. A microscope was used to observe the changes of the glomerulus and renal interstitium, and the images were collected. The results were confirmed by a pathological professional doctor.

### Transmission Electron Microscopy

The sections of the kidney tissues (1 mm^3^) were fixed in 2.5% glutaraldehyde for 2 h, followed by washing in 0.1 M phosphate buffer. After immersing in 1% osmic acid for 1.5–2 h, the kidney tissues were dehydrated through graded alcohols and immersed in an embedding medium overnight, and then the immersed sections were embedded in Epon 812 and dried in an oven. The dried kidney sections were cut into 40- to 60-nm slices and stained with uranyl acetate and lead citrate. A transmission electron microscope was used to examine the slices at 100 kV, and a CCD camera was used to take micrographs. The ultrastructure of the podocyte foot processes (FPs) was observed by using an electron microscope and the average width of the FP was measured to calculate the degree of fusion.

Foot process width was used to assess the podocyte effacement. For each TEM photograph, the glomerular basement membrane was traced and measured with standard image processing (Image-Pro Plus 6.0). The quantity of the FP overlying this part of glomerular basement membrane (GBM) was counted using Adobe Photoshop. The FP was defined as any connected epithelial segment butting on the basement membrane, between two neighboring filtration pores or slits. The arithmetic mean of the foot process width was calculated by using the following formula (Greka and Mundel, 2011; Qin et al., 2021):

$$The\;foot\;processs\;width=\left(\pi /4\right)\times \frac{\sum GBM\;length}{\sum quantity\;of\;foot\;process}$$

where Σ GBM length and quantity of the FP represented the total GBM length measured in one glomerulus and the total quantity of foot process counted, respectively. The correction coefficient π/4 was set to correct the random orientation in which the FP was sectioned.

### Radioimmunoassay

After plasma separation and podocyte supernatant collection, AngⅡ and CaN contents in the plasma and podocyte supernatant were determined by radioimmunoassay. We added a buffer, standard, quality control, and sample to be tested to each tube, then added markers and antibodies to incubate for 24 h. Finally, we add donkey anti-rabbit immunoskimmer to each tube and shake well, meanwhile placing it at room temperature for 15 min and measuring the radioactivity count of each precipitation tube.

### Immunohistochemical Microscopy

The kidney tissue fixed with 4% paraformaldehyde was taken out and prepared into sections, which were heated with citric acid for 2 min. 3% H_2_O_2_ was dropped on the tissue, and the tissue was incubated at room temperature. We then added the primary antibody, and the antibody ratio was TRPC6—1:200 and AT1R—1:400. The second antibody was added after incubation for 1 h, and then the DAB chromogenic agent was added, followed by hematoxylin staining, 1% hydrochloric acid ethanol differentiation, and lithium carbonate cyanidation in sequence. Finally, dehydration was performed for transparency.

### Cell Viability Assessment

Intervention of mature podocytes was performed with different concentration gradients of AngⅡ (1 × 10^−4^, 1 × 10^−5^, 1 × 10^−6^, 1 × 10^−7^, and 1 × 10^−8^ mol L^−1^) for 24 h. The MPC5 podocyte viability was assessed using CCK-8. The cells were seeded into 96-well plates, with three replicate wells for each group, at a density of 1 × 10^4^ cells per well, with 100 μl of medium. After the cells were incubated for the indicated time, 10 μl of the CCK-8 solution was added to each well, followed by incubation for 2 h. The optical density, OD, was computed at the absorbance of 450 nm, and the cell viability was calculated. According to the formula of “cell survival rate = (experimental group A value − blank group A value)/(normal group A value − blank group A value) ×100%" and “cell proliferation inhibition rate = (normal group A value − experimental group A value)/(normal group A value − blank group A value),” the results were calculated. We determined the optimal concentration of AngⅡ on the growth and proliferation of podocytes.

### Western Blotting Analysis

Kidney lysates were prepared with a RIPA lysis buffer containing phenylmethanesulfonyl fluoride (PMSF) and phosphate inhibitors (100:1:1). Equal amounts of selected protein extract samples were loaded onto sodium dodecyl sulfate–polyacrylamide gel electrophoresis (SDS-PAGE) gels and transferred to polyvinylidene difluoride (PVDF) membranes. After being blocked for 2 h in 5% skim milk/TBST, the PVDF membranes were washed with Tris-buffered saline containing 0.1% Tween-20 (TBST) thrice and incubated overnight at 4°C with different primary antibodies (TRPC6, 1:500; AT1R, 1:1,000; Caspase-3, 1:1,000; Nephrin, 1:1,000; and Podocin, 1:1000 corresponding to β-actin, 1:5000). Next, the PVDF membranes were washed with TBST thrice and incubated with horseradish peroxidase (HRP, 1:5,000)–conjugated secondary antibodies at room temperature for approximately 1 h. The immunoreactivity of the bands was visualized by Bio-Rad Image Lab with an electrochemiluminescence system. The densitometric analysis of the protein bands was accomplished by ImageJ 1.48 V and was normalized to relevant controls.

### Flow Cytometry

#### Apoptosis Rate Assay

The Annexin V–fluorescein isothiocyanate/propidium iodide apoptosis kit was used to detect apoptosis. First, the MPC5 podocytes were seeded at a quantity of 1 × 10^6^ cells/ml in a six-well plate. After each group was treated according to the experimental protocol, the cells were detached with 0.25% trypsin, gently triturated, and then washed with PBS after centrifugation. Additionally, the MPC5 podocytes were combined with Annexin V-FITC, PI, and 1 × buffer, and the solution was fully mixed and incubated in a dark room at 4°C for 30 min. A quantitative analysis of apoptotic cells was performed by flow cytometry. Flow Jo 7.6 software was used to analyze.

#### Fluorescence Intensity of Calcium Assay

The Fluo-3-AM mother solution was diluted into 3 μM Fluo-3-AM working solution using HBSS solution, and this was used to incubate the cells. Remove Fluo-3-AM working solution, added HBSS solution to incubate cells until completely de-esterify Fluo-3-AM. Fluorescence microscope was used to detect the fluorescence intensity of each group cell, and the excitation wavelength was 480 nm.

#### Statistical Analysis

SPSS 23.0 software was used to perform a statistical analysis on the experimental results, and data were reported as mean ± SD. Statistical evaluation was performed using a one-way analysis of variance (ANOVA) (two-sided test), followed by the least significant difference (LSD) (equal variances assumed) for the *post hoc* test, and also using the nonparametric test, namely, Mann–Whitney U-test, as a post-test. *p* < 0.05 indicated that the difference was statistically significant.

## Results

### *In Vivo* Study of Urinary Protein Content at 24 h

**FIGURE 1:**
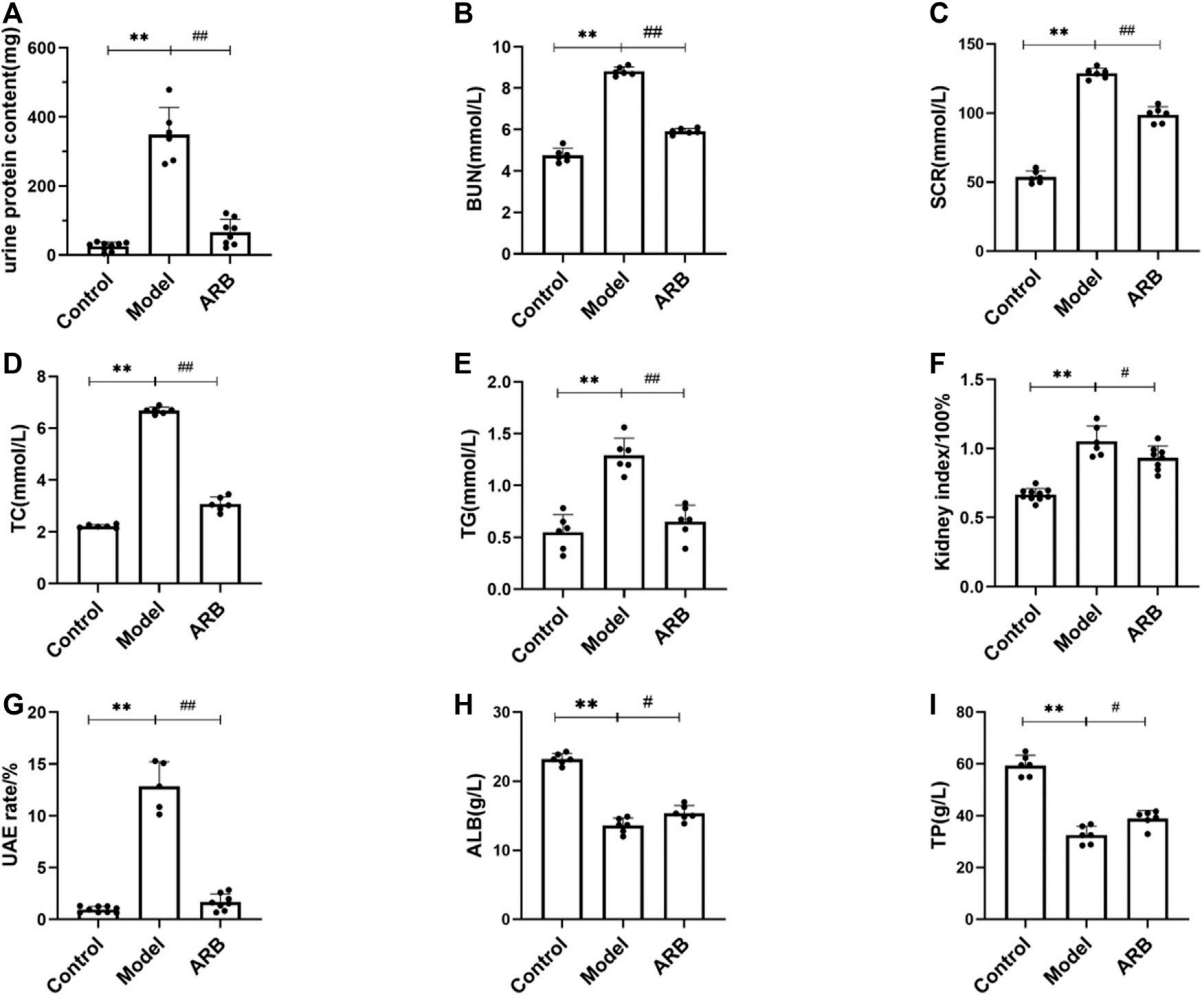
The serum and biochemical indexes of the three groups of rats after drug intervention were detected with an automatic biochemical analyzer. **(A)** 24-h urine protein content in the three groups of rats after 4 weeks. **(B)** BUN content in the three groups of rats after 4 weeks. **(C)** SCR content in the three groups of rats after 4 weeks. **(D)** TC content in the three groups of rats after 4 weeks. **(E)** TG content in the three groups of rats after 4 weeks. **(F)** (Kidney index = kidney weight/body weight) in the three groups of rats after 4 weeks. **(G)** UAE in the three groups of rats after 4 weeks. **(H)** ALB content in the three groups of rats after 4 weeks. **(I)** TP content in the three groups of rats after 4 weeks. (Compared with the control group, ^**^ *p* < 0.01; compared with the model group, ^##^ *p* < 0.01, ^#^ *p* < 0.05).

Two weeks after ADR administration, 24-h urine protein content of rats increased significantly when compared to the control group. At the end of the experiment, the urine protein content of the model group remained at a high level, suggesting that the modeling was successful. After 4 weeks of administration, we observed that the urine protein content in the ARB group was significantly lower than that of the model group (as shown in Figure 1A, *p* < 0.01) and tended to the control group value; the result showed that the ARB had a protective effect on proteinuria.

### *In Vivo* Effect of Angiotensin Receptor Blocker on Renal Dysfunction

Firstly, using the NS rat models induced by ADR, the kidney index, TG, TC, TP, proteinuria, ALB, and renal functional indicators such as BUN, SCR, and UAE were observed. As shown in Figure 1, in addition to TP and ALB, the increase in the values of urine proteinuria, BUN, SCR, TC, TG, UAE, and the kidney index were observed significantly 2 weeks after the induction of renal injury when compared to those of the control group rats. After induction of renal injury, the rats in the model group and the ARB groups showed ALB and TP loss to different degrees. At the end of 4 weeks after the treatment of ARB, ALB and TP were increased even faster when compared to those of the model group. Moreover, after the treatment of ARB for 4 weeks, the levels of urine proteinuria, BUN, SCR, TC, TG, and UAE and the kidney index in the ARB model rats were decreased significantly when compared to those of the model group rats (Figures 1A–I).

### Expression of AngⅡ and CaN *in Vivo* and *in Vitro*

**FIGURE 2:**
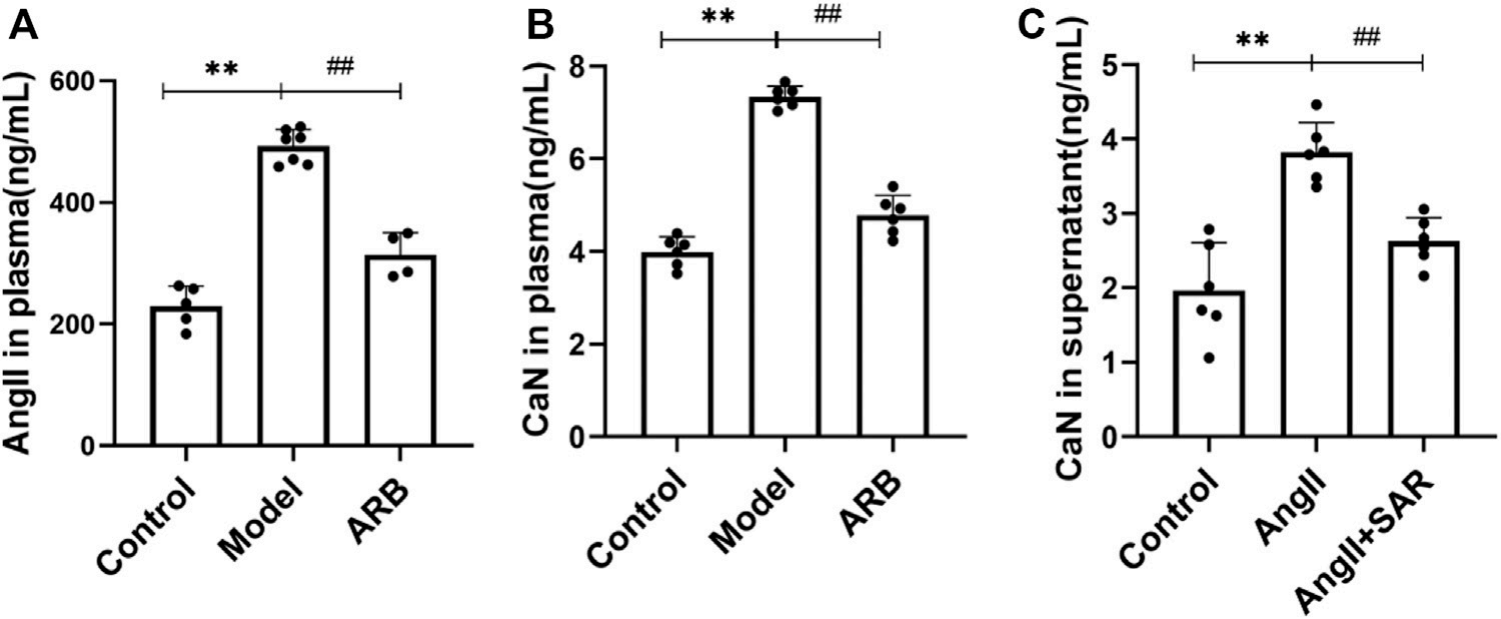
Detection of AngⅡ and CaN in the plasma and cell supernatant by using radioimmunoassay. **(A)** The content of AngⅡ in the plasma in the three groups of rats after 4 weeks. **(B)** The content of CaN in the plasma in the three groups of rats after 4 weeks. **(C)** The content of CaN in the cell supernatant after Ang II and TRPC6 inhibitor SAR treatment (Compared with the control group, ^**^ *p <* 0.01; compared with the model group, ^##^ *p <* 0.01, ^#^ *p <* 0.05).

We investigated the actions of the ARB and TRPC6 channel blocker SAR on the expressions of critical signaling molecules. As shown in Figure 2A, after the induction of renal injury, AngⅡ in the plasma level in the model group was increased obviously and decreased after 4 weeks of ARB drug intervention. Meanwhile, when compared with the control group, CaN content in the plasma and supernatant of podocytes after AngⅡ stimulation was significantly increased in the model group, while CaN content in the ARB group plasma and cell supernatant of the SAR group was significantly lower than in the model group (Figures 2B,C).

### *In Vivo* Study of Glomerular Structural Damage

**FIGURE 3:**
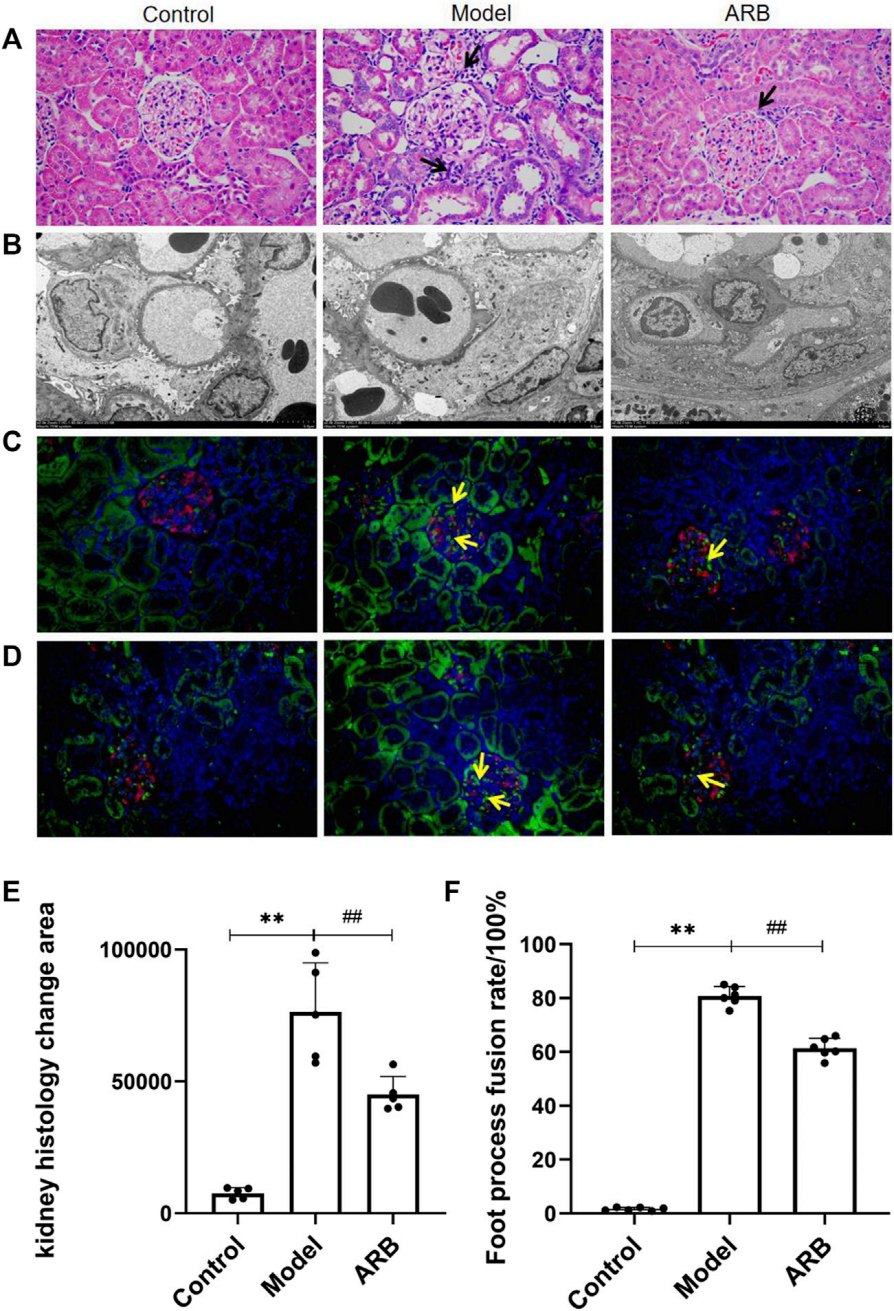
**(A)** Pathological pictures of the glomerular structure of the three groups of rats (×400). The black arrow indicates the injury sites of the glomerulus and renal tubules in the model group and ARB group when compared with the control group. The blue and purple cells are the nuclei, and the red cells are the cytoplasm. **(B)** Ultrastructural images in the three groups of rat podocytes by TEM. **(C)** Distribution of AT1R. Red signal is Nephrin, green signal is AT1R, and blue signal is the nucleus. Positive signals are marked with yellow arrows. **(D)** Distribution of TRPC6. The labeling method is the same as Figure **(C)**. **(E)** Quantification of kidney histology change. **(F)** The fusion rate of FP. The higher the fusion rate of FP, the more serious is the kidney damage. (Compared with the control group, ^**^ *p <* 0.01; compared with the model group, ^##^ *p <* 0.01, ^#^ *p <* 0.05).

After HE staining under a light microscope, it was found that the glomerular structure of the control group, without glomerular hypertrophy or atrophy, and the basement membrane thickness were uniform. Compared with the control group, the rats in the model group showed significant renal histopathological changes, such as proliferation of the glomerular mesangial matrix, capillary loop folding, thickening of the basement membrane, and deposition of a large number of immune complexes in the mesangial region and inflammatory infiltration. Compared with the model group, the aforementioned symptoms were significantly reduced in the ARB group (Figures 3A,E).

### Ultrastructural Changes of Podocytes and Distribution of Transient Receptor Potential Ion Channel 6 and AngⅡ Type I Receptor in Rat Renal Cortex *In Vivo*

Podocyte injury can induce proteinuria directly (Guo et al., 2014). Under the TEM, the podocytes in the control group were intact, FP was arranged clearly and orderly, there was no obvious podocyte fusion, and the basement membrane and endothelial cells were uniform. The model group podocyte structure was obviously discordant, and the fusion rate of FP was significantly higher than that of the control group. Compared with the model group, the aforementioned pathological manifestations were reduced in the ARB group, and the rate of FP fusion was reduced (Figures 3B,F). The distribution of AT1R and TRPC6 in the rats’ renal cortices are shown in Figures 3C,D; the green signal indicated by the yellow arrow is positive. We observed that AT1R and TRPC6 were found in traces and to be uniform in the renal cortex of the control rats. When compared with the control group, AT1R and TRPC6 in the model group were significantly increased. When compared with the model group, the aforementioned lesions in the ARB group were significantly reduced.

### Expression of AngⅡ Type I Receptor, Transient Receptor Potential Ion Channel 6, and Nephrin Proteins in Rat Renal Cortex *In Vivo*

**FIGURE 4:**
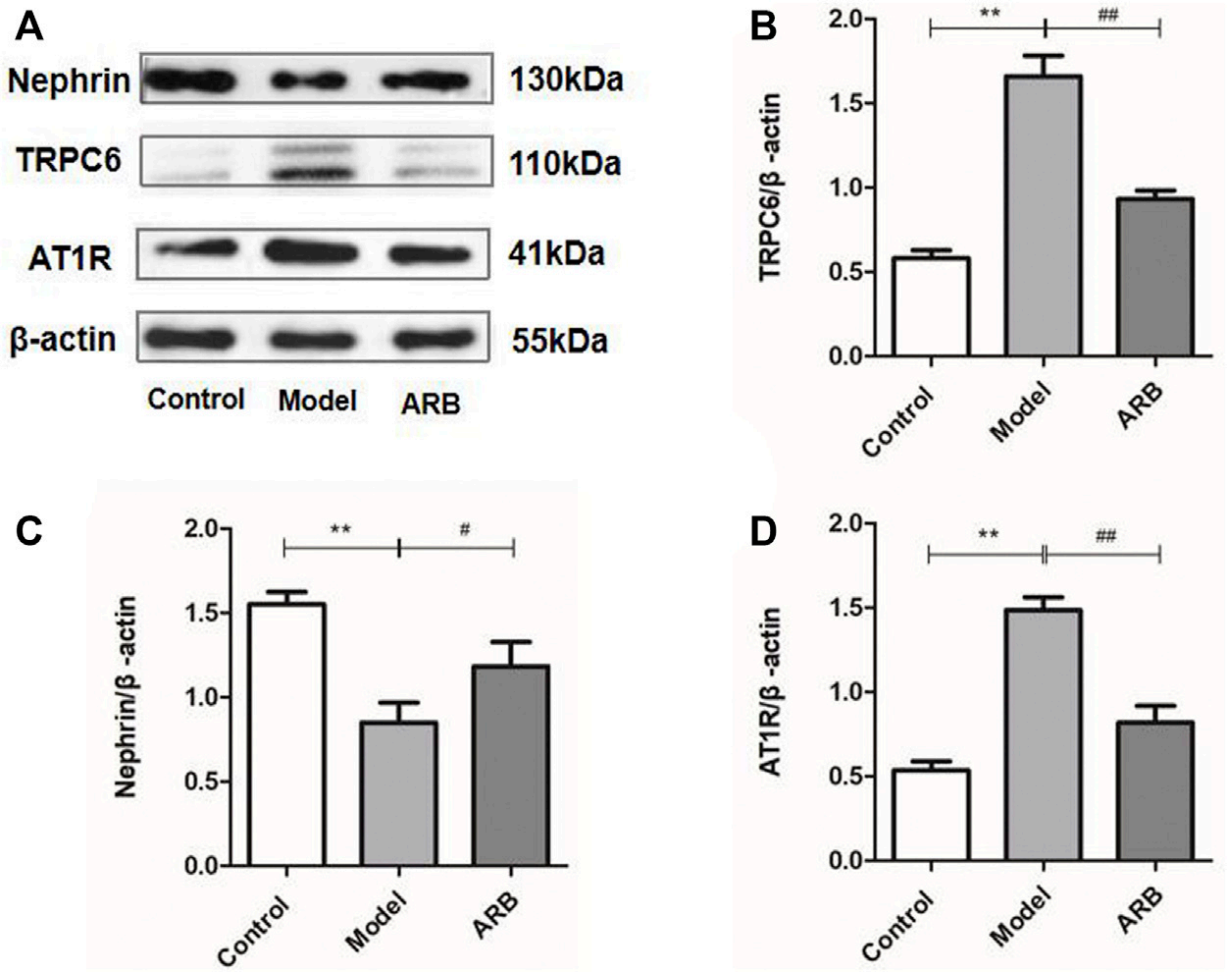
Relative expression of AT1R, TRPC6, and Nephrin proteins in the renal cortex in the three groups of rats. **(A)** Protein expression bands of AT1R, TRPC6, and Nephrin protein in the renal cortex in the three groups of rats. **(B)** Ratio of TRPC6 protein expression to internal reference β-actin. **(C)** Ratio of Nephrin protein expression to internal reference β-actin. **(D)** Ratio of AT1R protein expression to internal reference β-actin. (Compared with the control group, ^**^ *p <* 0.01; compared with the model group, ^##^ *p <* 0.01, ^#^ *p <* 0.05).

We investigated the expressions of AT1R, TRPC6, and Nephrin proteins in the renal cortex of the different groups of rats by the WB analysis. As shown in Figure 4, after ADR-induced kidney injury, the downregulated protein expression levels of Nephrin, and the upregulated protein expression levels of AT1R and TRPC6 in the kidneys of the model rats were detected. After the treatment of ARB for 4 weeks, the changed protein expression levels of AT1R, TRPC6, and Nephrin in the kidneys of the model rats were ameliorated obviously, when compared to those rats of the model group.

### Morphological Observation of Cells

**FIGURE 5:**
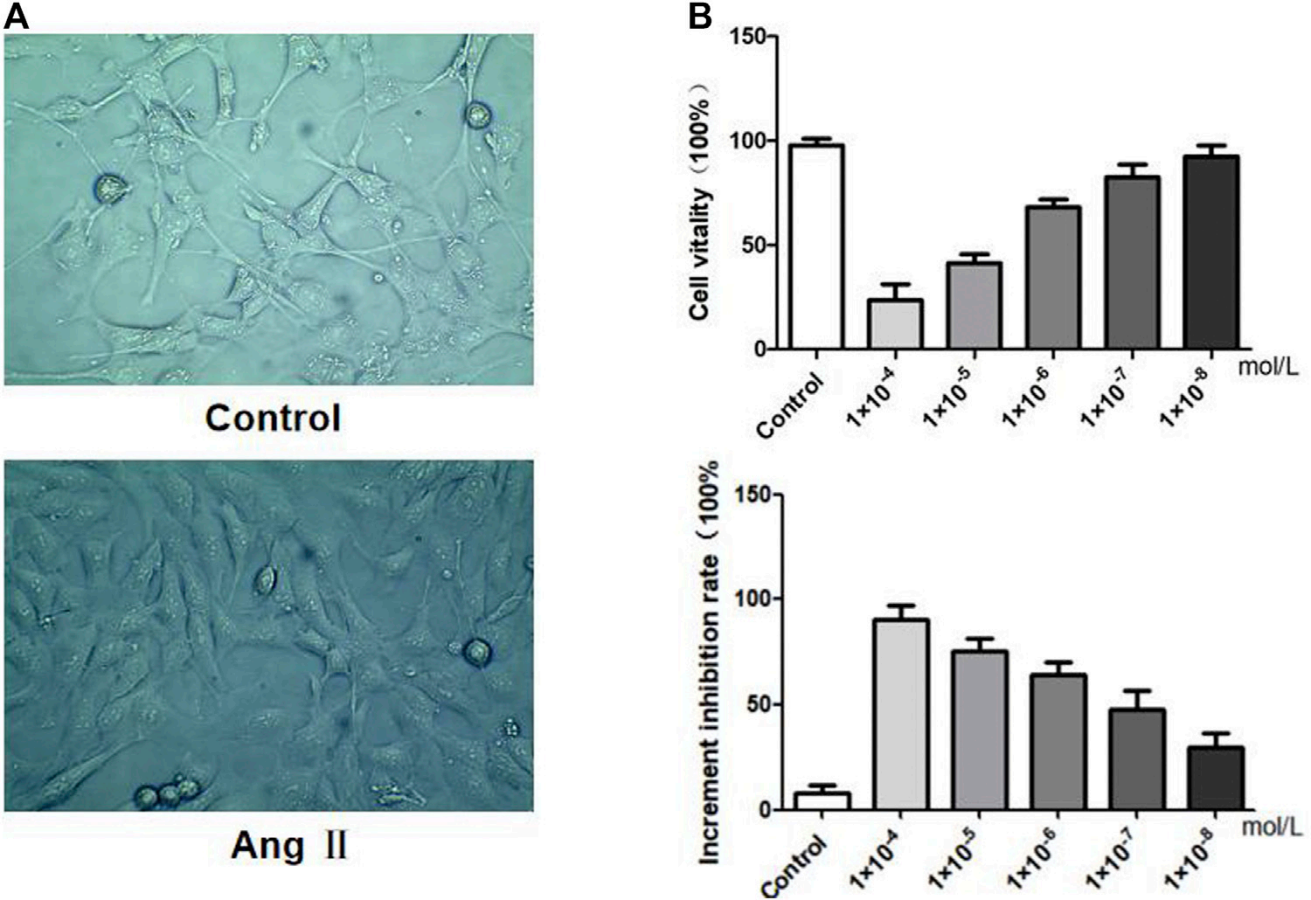
Effects of AngⅡ on MPC5 podocyte stimulation. **(A)** Morphological observation of podocytes before and after AngⅡ intervention. **(B)** Effects of AngⅡ concentration on podocyte viability and proliferation inhibitory rate.

As shown in Figure 5A, physiologically, podocytes have a distinct and long FP, and adjacent podocytes join with each other due to the linkage of FP. However, after exposure to AngⅡ, podocytes showed extensive fusion and significantly less intercellular contact, indicating that AngⅡ can directly damage podocytes. The results were consistent with our assumptions and previous animal experiments.

### Effects of AngⅡ on Morphology and Viability of MPC5 Podocyte *In Vitro*

We investigated the effects of AngⅡ on the morphology, *in vitro* viability and increased inhibition rate of MPC5 podocytes respectively. MPC5 podocyte morphology showed that control podocytes had long pseudopodia, while the pseudopodia retracted after AngⅡ intervention. The results of the cell viability assay indicated that when compared with the control group, the lower the concentration of AngⅡ on podocytes, the stronger was the cell viability. The inhibition rate of cell proliferation showed that the lower the concentration of AngⅡ on podocytes, the lower was the inhibition rate of proliferation. Therefore, we set 10^−6^ mol L^−1^ as the intervention level based on the activity and proliferation inhibition rate of different concentrations of Ang II on podocytes.

### Changes of Podocyte and Related Protein Expression Under Different Drug Intervention *In Vitro*

**FIGURE 6:**
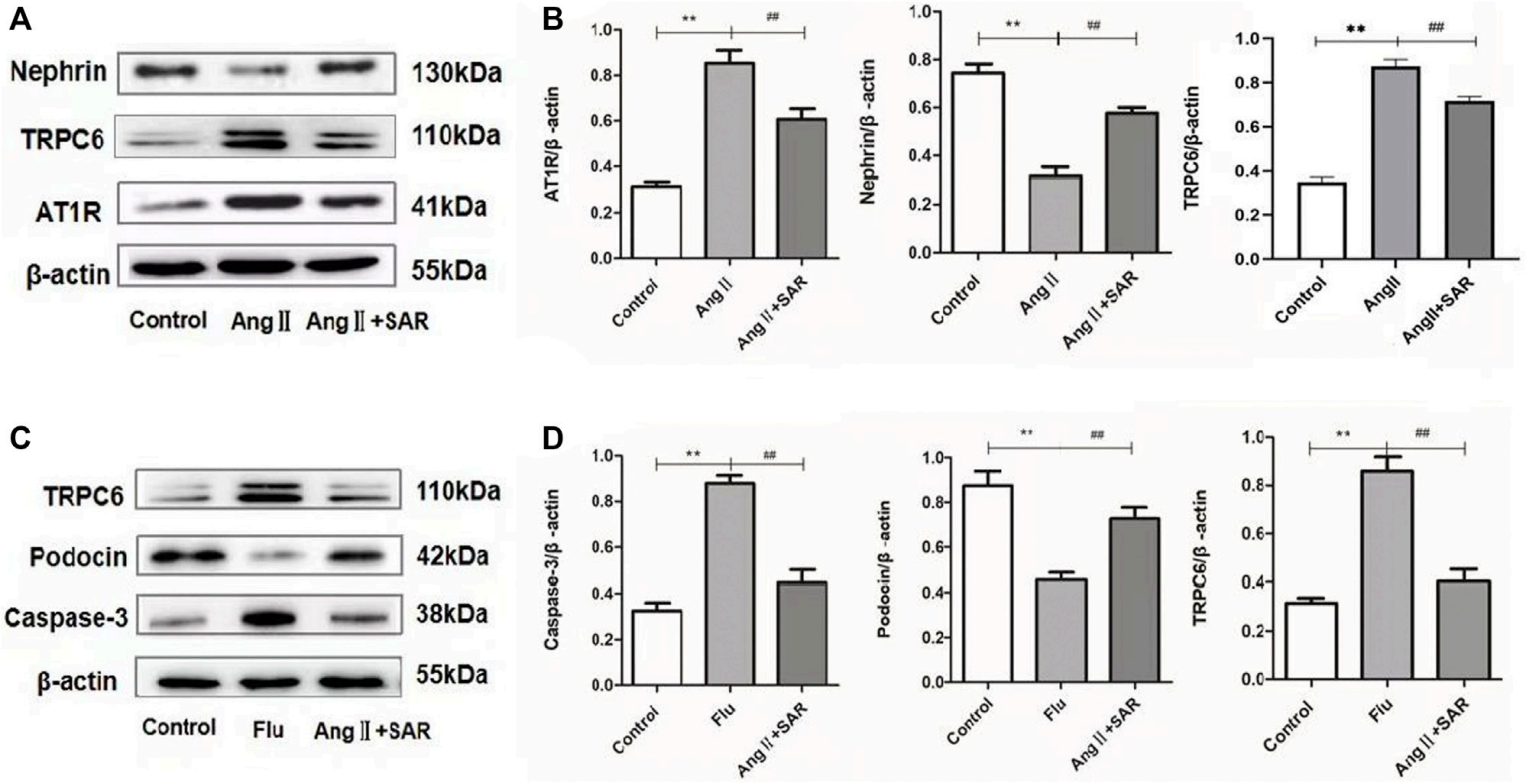
Relative expression levels of different proteins in podocytes. **(A)** Expressions of AT1R, TRPC6, and Nephrin in control, AngⅡ, and AngⅡ + SAR podocytes. **(B)** The ratio of AT1R, TRPC6, and Nephrin protein expressions to the internal reference β-actin. **(C)** Expressions of caspase-3, Podocin, and TRPC6 in control, Flu, and AngⅡ + SAR podocytes. **(D)** The ratio of caspase-3, Podocin, and TRPC6 protein expressions to the internal reference β-actin. (Compared with the control group, ^**^ *p <* 0.01, compared with AngⅡ/Flu group, ^##^ *p <* 0.01).

First, we confirmed the damage of AngⅡ to podocytes *in vitro*, and the TRPC6 inhibitor SAR had a certain protective effect on podocytes, as shown in Figure 6A. When compared with the control group, the expressions of AT1R and TRPC6 in the AngⅡ group (AngⅡ + normal podocyte) were significantly increased, and the expression of Nephrin protein was obviously decreased. On the contrary, the protein levels of AT1R and TRPC6 in the AngⅡ + SAR group were notably decreased, while the protein levels of Nephrin were tremendously increased when compared with the AngⅡ group. Subsequently, to test whether AngⅡ caused podocyte injury through TRPC6, we treated podocytes with TRPC6 agonist Flu and antagonist SAR, respectively. The experiment was divided into three groups, namely, the control, Flu (Flu + normal podocyte), and AngⅡ + SAR groups, as shown in Figure 6C. When compared with the control group, Flu also upregulated the expression levels of TRPC6 and Caspase-3 proteins after podocyte stimulations, while Podocin expression was significantly decreased, which was similar to the effect of AngⅡ on podocytes. Meanwhile, our results also showed that the use of TRPC6 inhibitor SAR could significantly reduce the expression levels of TRPC6 and Caspase-3 proteins and increase the expression levels of Podocin proteins when compared with the Flu group. Combined with the aforementioned experimental results, we can conclude that the effect of AngⅡ on TRPC6 is the same as that of Flu, the agonist of TRPC6. Therefore, we infer the upstream and downstream relationships between AngⅡ and TRPC6.

### Detection of Podocyte Apoptosis and Calcium Concentration in Podocytes by Flow Cytometry

**FIGURE 7:**
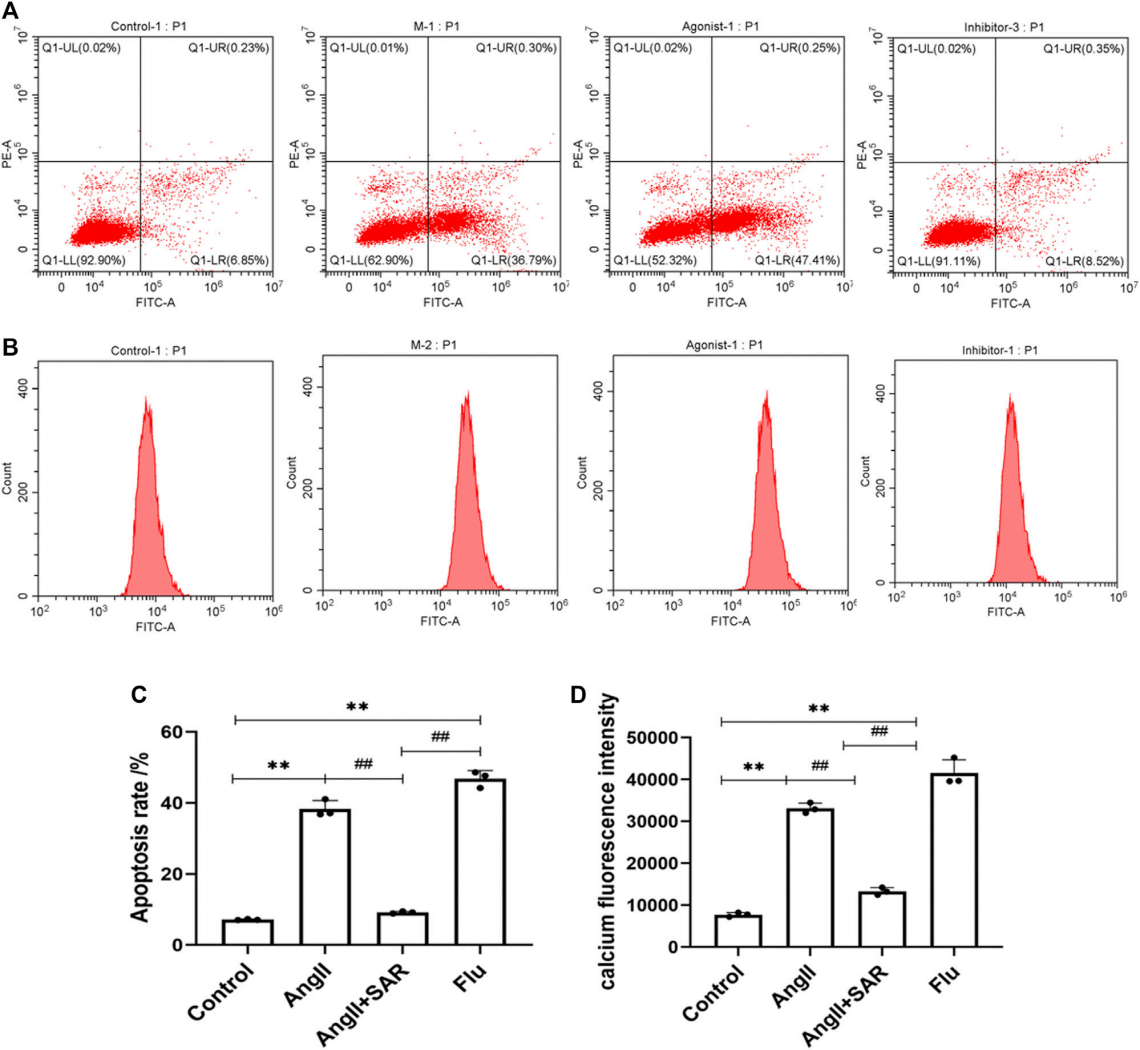
Differences in the podocyte apoptosis rate and calcium fluorescence intensity in normal and under different drug interventions of Ang, SAR, and Flu. **(A)** Differences in the podocyte apoptosis rate in normal and under different drug interventions of Ang, SAR, and Flu. Q1 was the necrotic cell, Q2 was the late apoptotic cell, Q3 was the early apoptotic cell, and Q4 was the number of viable cells. **(B)** Differences in calcium fluorescence intensity in normal and under different drug interventions of Ang, SAR, and Flu. **(C)** Quantitative plot of Figure **(A)**. **(D)** Quantitative plot of Figure **(B)** (Compared with the control group, ^**^ *p <* 0.01; compared with the AngⅡ group, ^##^ *p <* 0.01).

The apoptosis rate and fluorescence intensity of Ca^2+^ in podocytes in the different groups were detected by using flow cytometry. As shown in Figure 7A, when compared with the control group, the apoptosis rate of podocytes in the AngⅡ group and Flu was significantly higher and the early apoptotic cells were more obvious, indicating that the direct stimulation of AngⅡ can cause severe damage to podocytes by activating TRPC6, as does Flu, the TRPC6 agonist. Conversely, the apoptosis rate of the TRPC6 inhibitor SAR group was significantly lower than the AngⅡ and Flu groups. As shown in Figure 7B, the fluorescence intensity of Ca^2+^ in podocytes in the AngⅡ group and Flu were significantly higher than that in the control group, while the fluorescence intensity of Ca^2+^ in podocytes of the SAR group was significantly lower than that in the AngⅡ and Flu groups, even tending to normal. Therefore, the effect of the AngⅡ-TRPC6 axis on podocyte injury is associated with an increase in intracellular Ca^2+^ concentration, suggesting that Ca^2+^ responds to the downstream signaling of TRPC6.

## Discussion

NS is recognized worldwide as the leading cause of developing end-stage renal disease with severe renal dysfunction. The results showed that the renal function in model rats was significantly impaired. Especially, including the destruction of the glomerular structure, a large number of FP fusion. As a clinical symptom of NS, proteinuria is accompanied by the onset and development of NS. Proteinuria is mainly due to the destruction of the GFB, in which podocytes play an important role (Wang et al., 2021). Podocytes have free and dispersed FPs, and adjacent FP bridges to form slit diaphragm, dynamically modified by the actin cytoskeleton (Hu et al., 2020). The extensive actin cytoskeleton enables podocytes to dynamically contract under various mechanical stress stimulations, forming a tight network with cell adhesion molecules to stabilize glomerular filtration (Akchurin and Reidy, 2015). The loss of stress fibers and collapse and reorganization of the cytoskeleton are regarded as hallmarks of a podocyte injury (Kriz and Lemley, 2017; Sever and Schiffer, 2018). Consequently, we use podocytes as a good model system to study the physiological environment of calcium signaling and actin dynamics (Patrakka and Tryggvason, 2010). In this research, we selected the most representative podocyte structural proteins Nephrin and Podocin to study. WB results showed that the expressions of Nephrin and Podocin significantly decreased in model rats as well as in AngⅡ-stimulated podocytes. Kidney pathology further showed significant fusion or even the disappearance of podocytes, suggesting that the podocyte molecular barrier was destroyed.

The reduction of NS blood volume leads to an abnormal RAS activity, stimulates sympathetic nerve endings (Yue et al., 2015), and up-regulates the synthesis and secretion of AngⅡ. As the most biologically active substance in the RAS system (Li et al., 2017), AngⅡ can induce podocyte apoptosis *in vitro* and *in vivo* (Zhao et al., 2008). What’s more, clinical studies have found that AngⅡ levels are abnormally elevated in patients with nephropathy (Dryer et al., 2019). In animal experiments, an increased level of AngⅡ can induces hypertrophy of mesangial cells, leading to glomerular hypertension and elevated urinary protein (Yang et al., 2017). Similarly, recent studies found severe renal hypoxia, hypertension, proteinuria, and fibrosis in eSphK1−/− mice injected with AngⅡ (Xie et al., 2020). AT1R is the main receptor for AngⅡ in the kidney (Chi-Xianggeng et al., 2015). Nevertheless, AngⅡ is only biologically active when it recognizes and binds to its specific receptor (Muñoz et al., 2011). Although our results demonstrate the effect of losartan on AngII-TRPC6, as an Ang II receptor antagonist, losartan can improve hemorheology, renal hemodynamics, reduce renal vascular resistance, and reduce glomerular pressure, which may have a therapeutic effect on proteinuria. Whether losartan is hemodynamic-dependent, -independent or both requires further study.

TRPC6 is a channel protein located in the foot process of glomerular podocytes (Park et al., 2019), which regulates intracellular calcium signals (Asghar and Törnquist, 2020). Studies have shown that TRPC6 is a non-selective cationic downstream channel induced by AngⅡ elevation, and TRPC6 overexpression seems to be strongly correlated with the occurrence of glomerular proteinuria (Kalluri, 2006; Verheijden et al., 2018). Thus, we have identified TRPC6 as an important factor in initiating downstream podocyte injury. In animal experiments, we observed that the expressions of AngⅡ and its receptors AT1R and TRPC6 were up-regulated in the plasma and renal cortex of model rats compared with the control group. To verify the relationship among AngⅡ, AT1R, and TRPC6 in a podocyte injury, we stimulated MPC5 cells with AngⅡ. After the intervention of AngⅡ, the connections between the FPs were significantly weakened, and the FPs were widely fused in the podocytes. Moreover, WB results showed that the expression levels of TRPC6 and AT1R proteins were significantly increased, the expression levels of actin molecules were decreased, and apoptotic molecules were significantly upregulated, which was the same as the results of the TRPC6 agonist Flu. And simultaneously, the aforementioned indicators can be suppressed by SAR. Recent findings also support the possibility of cross-linkages among cytoskeletal regulatory protein molecules, slit diaphragm proteins, and podocyte Ca^2+^ signaling (Greka and Mundel, 2012), which is consistent with our results. Our results show that TRPC6 increases intracellular Ca^2+^ levels. In addition to enhancing intracellular Ca^2+^ levels, TRPC family members are reported to activate the small GTPases—RhoA and Rac1 (Tian et al., 2010; Greka and Mundel, 2011; Hall and Spurney, 2017). RhoA and Rac1 play key roles in regulating cytoskeletal dynamics in podocytes, and dysregulating the activity of these small GTPases in glomerular disease processes causes proteinuria. Rac1 and RhoA associate with and are activated by TRPC5 and TRPC6, respectively. Consistent with an important role for TRPC5 in glomerular diseases, inhibition of TRPC5 ameliorated glomerular injury in proteinuric rodent models (Zhou et al., 2017). Other members of the TRPC family have effects on glomerular diseases, such as TRPC5 and TRPC3. This study only discusses the role and mechanism of TRPC6, which is not comprehensive.

**FIGURE 8:**
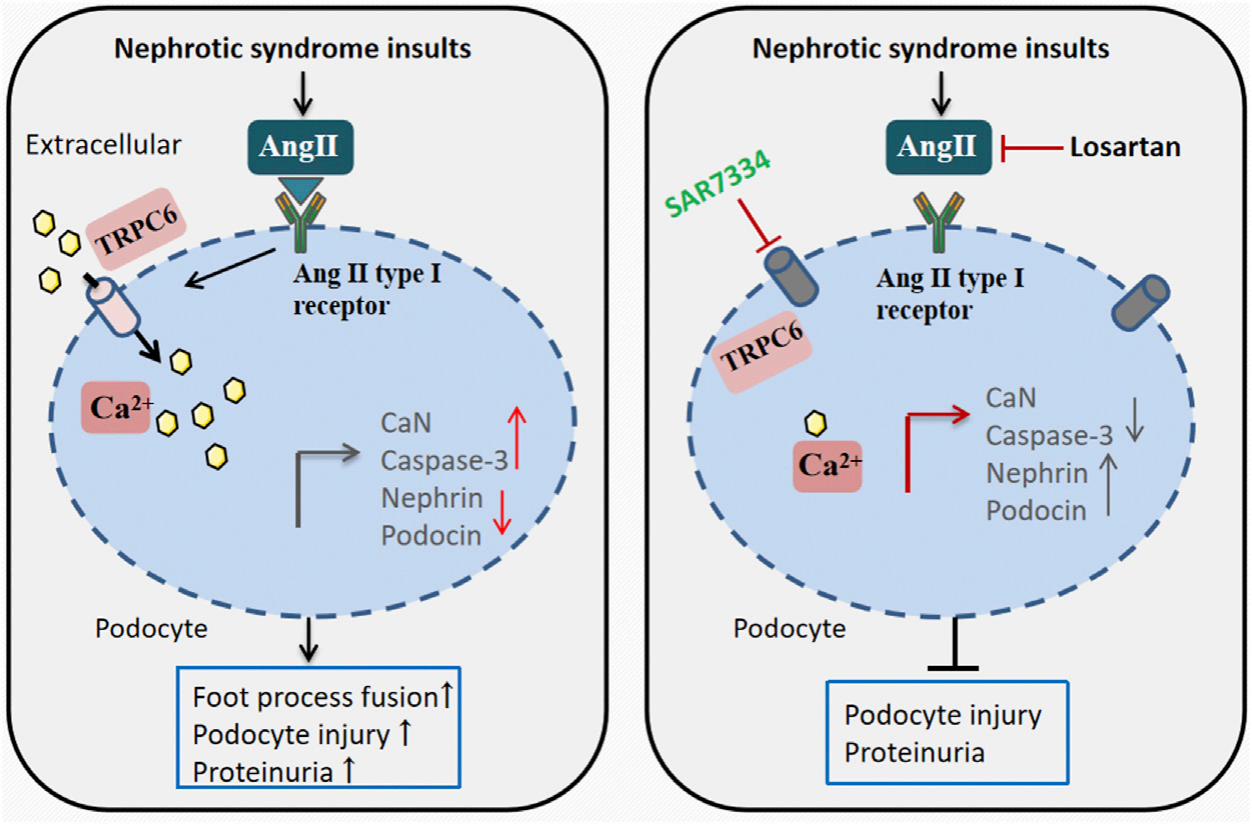
A working model for losartan and transient receptor potential cation channel 6 (TRPC6) antagonist SAR mitigating the NS-elicited podocyte injury *via* targeting the AngⅡ-controlled TRPC6 cellular calcium influx defense pathway. The TRPC6-dictated regulation of Ca^2+^ plays a key role in governing the injury response at a delayed/late phase. AngⅡ can catalyze TRPC6 activation. NS insults are capable of eliciting AngⅡ hyperactivity. This enhances AngⅡ-mediated TRPC6 activation, leading to CaN and apoptosis signal expression and actin cytoskeleton molecular degradation that results in podocyte injury, aggravating proteinuria. Losartan can directly inhibit AngⅡ activity, as well as SAR, inhibit TRPC6 activity, and thereby suppress TRPC6-mediated calcium influx at the same effect, mitigate the expression export of CaN and apoptosis signal, increase actin cytoskeleton molecular accumulation, and ultimately attenuate podocyte injury and proteinuria. CaN, calcineurin.

Podocyte injury is related to the disappearance of FP and rupture of the diaphragm on the one hand, and intracellular Ca^2+^ overload on the other (Coward et al., 2005; Castrop and Schießl, 2017). Ca^2+^ is an important regulator of cell homeostasis, regulating a variety of biological processes, such as muscle contraction, cytoskeletal structure, and programmed apoptosis (Staruschenko et al., 2019). Accumulating evidence suggests that Ca^2+^ signaling enhancement may play a significant role in the pathogenesis of podocyte injury (Gao et al., 2017; Chen et al., 2020). We found that the elevated levels of AngⅡ in podocytes can change the dynamic and stable state of Ca^2+^ in podocytes to an adaptive migration state, resulting in the imbalance of intracellular and extracellular Ca^2+^ (Kang et al., 2019). As a downstream effector of calcium signaling, CaN is a negative regulator of podocyte growth signal transduction. Activation of CaN in podocytes is sufficient to lead to the degradation of actin molecules and proteinuria (Rusnak and Mertz, 2000; Ilatovskaya et al., 2017). The results showed that the content of CaN in the plasma of NS rats was upregulated, but the AngⅡ receptor antagonist losartan could reverse the increasing trend of the plasma CaN level in NS rats. Meanwhile, in *in vitro* experiments, flow cytometry showed that AngⅡ and Flu could directly stimulate podocytes and significantly increase intracellular Ca^2+^ concentration. SAR acts by inhibiting intracellular calcium overload (Figure 8).

## Conclusion

In conclusion, *in vitro*, AngⅡ- and TRPC6-specific agonist Flu stimulated the expression of TRPC6 and AT1R in podocytes, accompanied by an increase in intracellular CaN and calcium overload. Losartan- and TRPC6-specific inhibitor SAR can effectively improve the overexpression of the aforementioned indicators. The results substantiated the possible mechanism of the AngⅡ-AT1R-TRPC6 axis mediating podocyte injury by promoting intracellular Ca^2+^ homeostasis imbalance and actin loss. Therefore, therapeutics targeting actin cytoskeletal regulatory molecules, calcium signaling, and apoptosis are potential research areas for the treatment of glomerular podocyte diseases. In addition, we also found that losartan can ameliorate Ca^2+^ overload to some extent. Of course, there are some problems in our study, such as not testing the related genes and ignoring the effects of losartan hemodynamics. Therefore, in the following research, we will use other techniques to find more direct evidence, such as RNA interference technology, patch clamp technology, etc.

## Data Availability

The original contributions presented in the study are included in the article/supplementary material; further inquiries can be directed to the corresponding authors.
